# Turning up the heat on skeletal muscle adaptations and neuromuscular function: key considerations for passive heating prescription and best practices

**DOI:** 10.1007/s00421-025-05917-9

**Published:** 2025-07-29

**Authors:** Patrick Rodrigues, Geoffrey M. Minett, Lucas B. R. Orssatto

**Affiliations:** 1 School of Sport and Human Movement, University of Waikato, Hamilton, New Zealand; 2 School of Exercise and Nutrition Sciences, Faculty of Health, Queensland University of Technology (QUT), Brisbane, Australia; 3 Centre for Sensorimotor Performance, School of Human Movement and Nutrition Sciences, The University of Queensland, Brisbane, QLD Australia

**Keywords:** Passive heating, Heat therapy, Hot-water immersion, FITT principles, Skeletal muscle adaptation, Neuromuscular function

## Abstract

Despite compelling evidence supporting the benefits of passive heat therapy in promoting skeletal muscle adaptation and enhancing neuromuscular function, the topic remains debated. Some recent studies report no significant effects on muscle protein synthesis, muscle mass, recovery, strength, or power. This raises critical questions: is passive heat therapy not actually effective? Or do these discrepancies reflect inconsistencies in study protocols and an overgeneralisation of the term *passive heating*? Despite its growing recognition as a health treatment, exercise mimetic, and tool for sport performance and recovery, the interpretation of outcomes is often simplistic or misinformed. In this opinion article, we discuss the disparities in the literature and highlight the risks of oversimplification, such as the binary view that passive heat therapy either “works” or “does not work.” We also propose incorporating the FITT (Frequency, Intensity, Time, and Type) principles from exercise science into thermal therapy research to enhance methodological consistency and clarity. By adopting a more structured and rigorous approach, the field can better realise the potential of passive heat therapy as a scientifically grounded intervention for both health and sport performance applications.

## Introduction

Passive heating refers to the application of external heat to the body, promoting physiological changes (e.g., increased heart rate, blood flow, and elevations in core, skin, and muscle temperature) without requiring active participation from the individual. Passive heat therapy (repeated heat exposure sessions) has been shown to enhance overall health, with compelling evidence showcasing its positive effects on the cardiovascular, respiratory, metabolic, neurological, and musculoskeletal systems (Brunt and Minson 2021; Ely et al. 2017; Hunt et al. 2020; Pizzey et al. 2021). It has also been suggested to extend health and lifespan (Patrick and Johnson 2021; Laukkanen and Kunutsor 2024). Considering these benefits, passive heating is often termed as an "exercise mimetic," because it triggers overlapping physiological pathways (e.g., cardiovascular, metabolic, neurological, and musculoskeletal systems) similar to those stimulated by moderate-intensity exercise. Thus, it offers a promising alternative for individuals who are unable to engage in physical activity or have low exercise tolerance.

Musculoskeletal adaptations from passive heat therapy include muscle hypertrophy, accelerated muscle recovery following induced muscle damage, and muscle atrophy prevention during periods of disuse (Hafen et al. 2019; Rodrigues et al. 2020a; Goto et al. 2011; Normand-Gravier et al. 2025). These responses are associated with enhanced heat shock protein (HSP) expression, mitochondrial function, and skeletal muscle cell metabolism (Kim et al. 2020a; Pallubinsky et al. 2020; Marchant et al. 2023), all triggered by passive increases in muscle temperature. Regarding neuromuscular function, acute passive heating has been shown to enhance rapid muscle contraction (Mornas et al. 2021; Morrison et al. 2004; Périard et al. 2014; Racinais et al. 2017; Rodrigues et al. 2021, 2023; Thomas et al. 2006), while repeated sessions improve maximal voluntary contraction (MVC) (Goto et al. 2011; Racinais et al. 2017; Kim et al. 2020b). However, despite compelling original evidence and reviews (Rodrigues et al. 2020a, 2022; Kim et al. 2020a; Normand-Gravier et al. 2025) extensively discussing the acute and chronic effects of passive heating on skeletal muscle adaptation and neuromuscular function, the topic remains debated. Some recent studies have reported and concluded that passive heat therapy has no significant effects on muscle protein synthesis, muscle mass, recovery, strength, or power (Labidi et al. 2021; Fuchs et al. 2020, 2024; Gustafsson et al. 2025). Hence, the question arises: why is passive heat therapy no longer effective? In our opinion, these discrepancies may not reflect a failure of this type of intervention itself, but instead, stem from heterogeneity in study protocols combined with the generalisation of the term “passive heating.”

Despite the growing recognition of passive heating interventions as a health treatment, exercise mimetic, or a tool for sport performance and recovery, the interpretation of its outcomes is often simplistic and overlooked. Unlike exercise interventions, where careful attention is given to variables such as frequency, intensity (internal and external loads), volume (duration or number of sets and repetitions), and type (e.g., aerobic, anaerobic, resistance, or concurrent training) before any conclusion is drawn, passive heating research often lacks comparable rigor when interpreting its outcomes, inadequately generalising the study findings. This oversimplification limits the precision of its application and diminishes its scientific validity. If passive heat therapy indeed offers health benefits, exercise mimetic effects and sport performance outcomes, why do we neglect the same level of precision, application, and interpretation in its interventions? It is time for a paradigm shift—one that challenges the notion that all passive heating interventions are interchangeable and establishes a framework for prescribing passive heating with the same methodological rigor as exercise in its prescription and interpretation.

This opinion paper emphasises avoiding generalisations in passive heat therapy research. It proposes guidelines for reporting and describing passive heating protocols and recommends best practices to optimise musculoskeletal adaptations and neuromuscular function improvements. By adopting a more structured approach, the field can unlock the full potential of passive heat therapy as a scientifically robust intervention in both health and sport contexts.

## The effects of passive heat therapy on muscle mass, recovery and neuromuscular function

Although the beneficial effects of passive heating on skeletal muscle adaptation, recovery and neuromuscular function are well documented, the minimal and optimal dosage (endogenous and exogenous thermal stress/strain) remain poorly defined and not well established. Recent studies (Labidi et al. 2021; Fuchs et al. 2020, 2024; Gustafsson et al. 2025) have suggested that passive heat therapy does not influence muscle mass, strength or recovery, contrasting with previous evidence (see Rodrigues et al. 2020a, Kim et al. 2020a and Normand-Gravier et al. 2025 for reviews). These conflicting statements can lead to confusion and misinterpretations of the effects of passive heat therapy. Hence, a closer examination of the passive heating protocols is warranted to reconcile these discrepancies.

Labidi et al. (2021) investigated the effects of 6 weeks of localised heat therapy on muscle mass, strength and contractile properties (e.g., peak twitch torque) in physically active, healthy adults. Participants used adhesive heating pads on the gastrocnemius muscle for eight hours a day, five times a week. The protocol was based on the study of Goto et al. (2011), who reported increases in isometric maximal force and muscle mass (quadriceps and single muscle fibre cross-sectional area) after 10 weeks of localized heat therapy (8 h/day, 4 days/week) in healthy middle-aged men (45 ± 6 years). Despite the similar duration and volume of the protocol, differences in passive heat intensity may explain the contrasting findings. Labidi and colleagues observed muscle temperature increases to ~37.3 °C, while Goto and colleagues reported ~38.3 °C. Additionally, differences in the target muscle (gastrocnemius vs. quadriceps) and participant age (35 ± 6 vs. 45 ± 6 years) could partially account for the divergent outcomes. These findings highlight the importance of avoiding generalisations in passive heat therapy research, as a ~1 °C difference in muscle temperature may significantly impact results.

Following eight weeks of intervention, Fuchs et al. (2024) concluded that passive heat therapy does not affect muscle hypertrophy (e.g., fibre size and cross-sectional area) or muscle strength in healthy older adults (73 ± 6 years). Participants underwent 45-min infrared sauna sessions at 60 °C three times per week. However, the study did not report core or muscle temperature, making it difficult to assess the thermal strain's intensity or accumulation (i.e., the area under the “intensity × duration interaction” curve). In contrast, an earlier study (Racinais et al. 2017) demonstrated increases in MVC torque  and peripheral muscle contractility (i.e., evoked contractions) after eleven days of whole-body heat stress. In this study, participants were exposed to heat stress in an environmental chamber at 44–50 °C and 50% relative humidity (RH), targeting a core temperature of 39 °C for one hour. Together, these findings suggest that the absence of improvements in the studies of Labidi et al. (2021) and Fuchs et al. (2024) may result from insufficient endogenous (internal) thermal strain (intensity and duration) when compared to early reports (Hafen et al. 2019; Rodrigues et al. 2020a; Goto et al. 2011). Specifically, raising muscle temperature only to ~37.3 °C or undergoing a short sauna session at 60 °C may not provide the necessary stimulus for muscle mass and neuromuscular adaptations.

In the context of passive heat therapy for muscle recovery, Gustafsson et al. (2025) applied lower-limb hot-water immersion at 42 °C for 20 min following a 90-min simulated soccer match. They found no improvement in post-match performance recovery, assessed via sprinting, jumping, fatigue and MVC tests. However, the short duration of the protocol may have been insufficient to elicit physiological recovery benefits, and their conclusions appear to overgeneralise their findings across all hot-water immersion approaches. In contrast, Sautillet et al. (2024) used a 45-min lower-limb immersion following eccentric muscle damage, comparing water temperatures of 40 and 41 °C. Only the 41 °C condition significantly improved muscle recovery, suggesting a dose–response relationship where even a 1 °C difference can affect outcomes. Similarly, Dablainville et al. (2025) applied 60 min of lower-limb immersion at 42 °C after muscle damage induced by electrically stimulated eccentric contractions, reporting reduced muscle soreness, lower creatine kinase and myoglobin levels, and increased both HSP synthesis alongside a shift in cytokine profiles. Despite differences in muscle damage methods and recovery measures, these studies collectively highlight the importance of protocol specificity and avoidance of generalisations. Both the intensity and duration, as well as their interaction (area under the curve), must be carefully considered when evaluating the efficacy of passive heat therapy. Notably, Sautillet et al. (2024) proposed that “maintaining core temperature (gastrointestinal) between 38.5 and 39 °C for approximately 25 min mitigates muscle fatigue”. However, these results may conflate core and muscle temperature effects. It is more likely that the observed benefits were primarily due to higher increases in local muscle temperature during 41 °C hot-water immersion, rather than core temperature. If core temperature alone were the key determinant, similar recovery outcomes should result from any modality achieving that threshold, regardless of which muscle groups are heated. This assumption risks misattributing recovery effects and overextending findings to unrelated muscle areas. For example, it would suggest that upper-limb recovery (e.g., elbow flexors) could be expected from lower-limb immersion, solely based on achieving the same core temperature and passive heating duration.

In summary, the effectiveness of passive heat therapy depends on achieving sufficient thermal stress through appropriate intensity, duration, and localisation to elicit meaningful musculoskeletal adaptations, neuromuscular improvements, and muscle recovery. Muscle temperature likely plays a more critical role than core temperature in driving these effects.

## Applying the FITT principles in passive heat therapy prescription and research

**Fig. 1:**
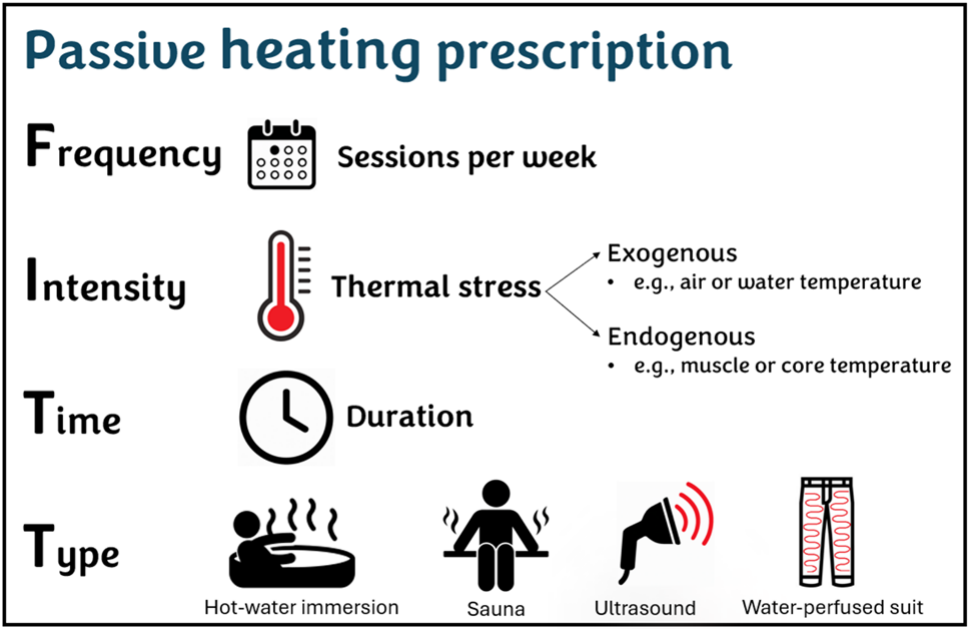
Passive heat therapy prescription based on FITT principles

Passive heat session prescriptions should be as meticulously detailed as exercise programs, and their effectiveness should be evaluated and interpreted according to the principles of Frequency, Intensity, Time, and Type (FITT) (Fig. 1). While FITT principles are well-established in exercise science, their adoption in thermal therapy research remains inconsistent. Incorporating these principles can enhance result interpretation, improve conclusions, and inform effective prescription and clinical applications.

While most studies detail the frequency, intensity, duration (time), and type (e.g., hot-water immersion, sauna, ultrasound, water-perfused suits) of passive heating protocols, their interpretation and conclusions often lack clarity and do not sufficiently account for each of these components. Additionally, the intensity of the passive heating should consider both external thermal stress (e.g., water or air temperature, relative humidity, shortwave pulses) and internal thermal strain (e.g., skin, core and muscle temperature) (see Table 1 for examples). Like exercise prescription, which considers external load (e.g., running speed or cycling power) and internal load (e.g., the volume of oxygen uptake [VO_2_] or heart rate). Addressing these factors can provide a comprehensive framework for designing and evaluating the effect of passive heating interventions.

**Table 1:** Examples of studies detailing passive heat therapy protocols based on the FITT principles

References	Frequency	Intensity	Time	Type
Hafen et al. (2019)	Ten consecutive sessions	Exogenous: 800 pulses per sec, pulse duration 400 μs Endogenous: Intramuscular temp, ↑ ∆4.2 ± 0.3 °C	2-h sessions	Pulsed shortwave diathermy
Brazaitis and Skurvydas (2010)	Seven sessions every second day, 14-day intervention	Exogenous: Water at 44 °C Endogenous: Rectal temp, Resting: 37.5 ± 0.1 °C After hot bath: 39.5 ± 0.1 °C	45-min sessions	Lower limbs hot-water immersion
Kim et al. (2020b)	5× a week, 8-week intervention	Exogenous: Circulating water at 52 °C Endogenous: Skin thigh temp, Control: 32.4 ± 0.3 °C Passive heating: 39.8 ± 0.3 °C	90-min sessions	Water-perfused suits
Racinais et al. (2017)	Eleven consecutive sessions	Exogenous: Room temp at 48–50 °C and 50% RH Endogenous: Rectal temp targeting 39 °C	1-h sessions	Environmental chamber
Goto et al. (2011)	4× week, 8-week intervention	Exogenous: Not reported Endogenous: Intramuscular temp, Resting: 34.9 ± 0.5 °C After 3 h: 38.2 ± 0.1 °C After 6 h: 38.3 ± 0.1 °C	8-h sessions	Local heating: A heat- and steam-generating sheet placed on the thigh laterally

### Thermal strain responses and different types of passive heating approaches

Whether whole-body or localised passive heating methods are used, the extent of internal muscle temperature elevation appears to be a key factor in eliciting mitochondrial and muscle cell adaptations (Marchant et al. 2023). Hence, localised heating is particularly advantageous when targeting musculoskeletal tissue, as it enables the application of higher heat stress to specific body segments (e.g., legs) while the rest of the body remains unaffected, thereby avoiding unnecessary increases in core temperature. In fact, several studies have reported mitochondrial and muscle cell adaptations following local passive heating without significant changes in core temperature (Goto et al. 2011; Hafen et al. 2018, 2019; Kim et al. 2020a, b; Gibson et al. 2023). Nevertheless, increases in core temperature may still play a role in driving neuromuscular adaptations (see section below).

Common passive heating interventions include hot air (saunas and environmental chambers), hot-water immersion, water-perfused suits, and localized heating by heating pads or pulsed ultrasound (microwave diathermy) devices. Each approach confers different physiological responses in core, skin and muscle temperatures.

**Fig. 2:**
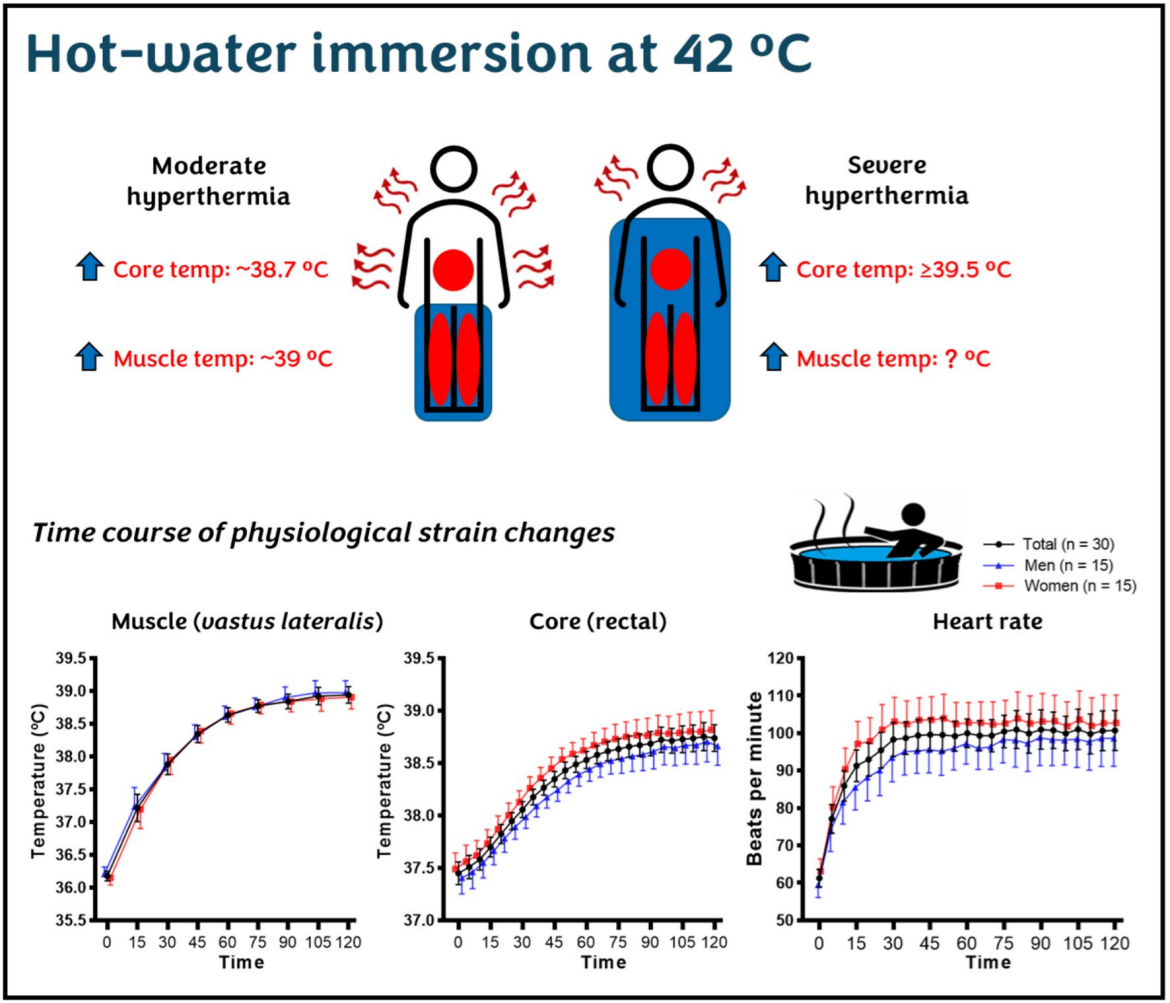
Conceptual illustration of thermal strain responses and time course of changes from a 42 °C hot-water immersion session. The *top figure* illustrates two individuals immersed in 42 °C hot water, one to waist level and the other to shoulder level. A waist-immersed individual would experience *moderate hyperthermia* (~38.7 °C) after ~95 min, with muscle temperature increasing up to ~39 °C. In contrast, an individual immersed up to the shoulders would reach *severe hyperthermia* (≥39.5 °C) due to the reduced surface area available for heat dissipation. However, the corresponding muscle temperature at a core temperature of 39.5 °C is unclear. These values are based on previous publications (Rodrigues et al. 2020b, 2025; Brunt et al. 2016; Tan et al. 2024). The *bottom figure* presents the time course of physiological strain during a 120-min hot-water immersion at 42 °C in 30 healthy adults (15 men, 15 women). The data show changes in muscle and core temperature, and heart rate responses over time, based on previous findings (Rodrigues et al. 2020b)

Hot-water immersion and water-perfused suits offer controlled heating of localised muscles while managing overall body temperature. For example, immersing the body in hot water to the waist exposes the lower limb muscles to heat stress while the surface area of the upper body remains free from heat, in contact with cooler air, allowing heat dissipation. If the water level rises to the chest or shoulders, the thermal stress to the lower limbs remains unchanged; however, the reduced surface area exposed to cooler air decreases heat dissipation, increasing core and mean skin temperature (Fig. 2). Hot air can be efficient if it aims to rapidly increase core and skin temperatures as it involves whole-body exposure. Therefore, when selecting the passive heating "type" approach, it is essential to consider whether the intervention aims to stress localised muscle cells or to induce systemic heat strain.

### Time course of physiological strain changes during passive heating sessions

Understanding physiological strain kinetics is crucial for an effective passive heating prescription. For instance, during a hot-water immersion at 42 °C up to waist level, muscle temperature takes ~85 min to plateau and reach peak, at ~ 39 °C, after 105 min. In comparison, core temperature takes ~95 min to plateau, at ~38.7 °C, with no futher increases and heart rate ~30 min to plateau maintaining at ~100 bpm (Rodrigues et al. 2020b) (Fig. 2). However, if the water level is raised to the shoulders, thereby reducing the surface area available for heat dissipation, core temperature will rapidly rise. Consequently, it is unclear whether muscle temperature reaches a plateau before severe hyperthermia (≥39.5 °C), or what the exact muscle temperature is when core temperature reaches 39.5 °C. This uncertainty is related to the high inter-individual variability in the time required to reach a target core temperature during heat exposure (Todd et al. 2005; Morrison et al. 2004; Périard et al. 2014; Racinais et al. 2017; Brunt et al. 2016). Therefore, these timelines emphasise the need to consider how long participants remain at target thermal strain levels (e.g., core and/or muscle temperature, heart rate) to achieve desired outcomes.

Previous research highlights the importance of carefully considering passive heating protocol designs. Brunt et al. (2016), for instance, aimed to maintain a core temperature between 38.5 and 39 °C for 60 min to study the effects of passive heating therapy by hot-water immersion on cardiovascular function. To accelerate the rate of core temperature elevation, participants were initially submerged to the shoulders in 40.5 °C water until their core temperature reached 38.5 °C (~25–35 min), then the water level was lowered to the waist for the remaining session to maintain thermal strain. In a similar design, but using hot air and studying neuromuscular function outcomes, Racinais et al. (2017) exposed participants to 50 °C (50% RH) in an environmental chamber until the core temperature reached 39 °C, then the air temperature was reduced to 44 °C, maintaining the physiological strain. Importantly, both studies observed positive results after the passive heat therapy. This underscores the importance of monitoring and adjusting external and internal thermal stress to achieve desired outcomes.

When designing repeated passive heating interventions (>5 sessions), it is essential to distinguish between heat stress (external thermal load) and heat strain (internal physiological response). Over time, thermal adaptations (e.g., reductions in core temperature and heart rate, and increased sweating rate) can cause these responses to diverge. For example, after seven sessions of lower-limb immersion at 44 °C for 45 min, post-immersion rectal temperature decreased from 39.5 ± 0.1 °C to 39.2 ± 0.1 °C (Brazaitis and Skurvydas 2010). Similarly, 6 weeks of daily post-exercise immersion at 42 °C (~45 min, up to the sternum) saw rectal temperature decreased from 39.2 ± 0.2 °C to 38.5 ± 0.4 °C post-immersion (Rodrigues et al. 2025). In an environmental chamber study, 11 days of heat acclimation (48–50 °C and 50% RH, 1-h sessions), increased the time required to reach a target rectal temperature of 39 °C from 59 ± 15 to 68 ± 13 min (Racinais et al. 2017). However, whether muscle temperature adapts similarly remains unknown. Thus, if passive heating protocols are guided primarily by endogenous intensity, within-intervention assessments during a multi-week protocol are recommended to adjust exposure intensity and/or duration.

That said, we acknowledge that quantifying thermal stress and strain is inherently complex. Applying thermodynamic principles to physiological responses and accurately measuring heat storage and exchange requires more than core and muscle temperature measurements (Taylor et al. 2014). Tissue temperatures are influenced by local metabolism, tissue conduction, and blood flow, and no single gold-standard measure exists. Core temperature sites (e.g., auditory canal, rectal, oesophageal, and gastrointestinal) exhibit different response kinetics during heat exposure (Taylor et al. 2014). Muscle temperature responses are also variable depending on individual muscle characteristics, subcutaneous fat thickness, and the measurement site and depth (Rodrigues et al. 2024). Therefore, body temperature measurements (whether core or muscle) should be based on the study aims. However, despite these challenges, studies should at a minimum report core and/or muscle temperature to support a meaningful interpretation of thermal strain.

In summary, by incorporating FITT principles into passive heat therapy research, future studies can design targeted interventions and improve reporting clarity. This approach will help delineate the nuances of passive heating and its effects on different physiological systems and diverse populations. While passive heat therapy is considered an exercise-mimetic strategy, it often requires longer sessions than exercise to induce significant responses due to slower kinetics of physiological changes under heat stress. Indeed, evidence suggests that longer sessions [60–120 min (Racinais et al. 2017; Rodrigues et al. 2021; Hafen et al. 2018; Dablainville et al. 2025)] are more effective than shorter ones [20–45 min (Labidi et al. 2021; Fuchs et al. 2024; Tan et al. 2024; Gustafsson et al. 2025)].

## Passive heat therapy for musculoskeletal adaptation and neuromuscular function

Increases in muscle temperature primarily drive skeletal muscle adaptations to passive heating and seem to follow a dose-dependent relationship (Obi et al. 2017, 2018; Sugi and Tsuchiya 1988; Sugi et al. 1992). Heat shock proteins (HSPs), a family of molecular chaperones, play a central role in the mitochondrial and cellular adaptations that occur in response to heat stress (Marchant et al. 2023). Passive heating elevates muscle temperature, which stimulates the synthesis of HSPs, triggering a downstream inflammatory cascade associated with muscle growth (Rodrigues et al. 2020a). In summary, it has been suggested that acute elevations in muscle temperature lead to inhibition of nuclear factor-ĸB (NF-ĸB) through the upregulation of HSPs, increasing 5′ AMP-activated protein kinase (AMPK) activity, and promoting activation of the protein kinase B (Akt)–mechanistic target of rapamycin (mTOR) signalling pathway, and greater expression of mitochondrial genes, as well as proliferation of muscle satellite cells (Rodrigues et al. 2020a; Marchant et al. 2023; Hafen et al. 2018; Fennel et al. 2023; Goto et al. 2011; Kim et al. 2020a). Furthermore, AMPK and Akt–mTOR signalling pathways suppress muscle atrophy-related mechanisms that promote myofibrillar protein degradation (Bodine and Baehr 2014; Hafen et al. 2018, 2019). However, when muscle temperature exceeds a threshold (e.g., 42 °C), heat exposure promotes protein degradation over synthesis (Baracos et al. 1984; Luo et al. 2000; Essig et al. 1985), impairing musculoskeletal adaptations. Thus, understanding muscle temperature kinetics during passive heating interventions is essential for optimising outcomes and establishing mechanistic links between thermal exposure and mitochondrial and skeletal muscle cell adaptations.

Microwave diathermy devices are among the most efficient methods for increasing muscle temperature. They can rapidly elevate muscle temperature and plateau in 30 to 40 min (Hafen et al. 2018), reaching up to 40 to 41.5 °C (Hafen et al. 2019; Nosaka et al. 2007). However, these devices are limited to heating single muscles each time, whereas hot-water immersion and hot air can target multiple muscles per session.

Hot-water immersion is generally more effective at raising muscle temperature than hot-air methods due to a higher heat conduction rate, approximately 24 times greater than air. This allows for faster increases in muscle temperature, particularly in deeper muscles (e.g., soleus, vastus intermedius), which exhibit lower temperature variations than superficial muscles (Rodrigues et al. 2024). In addition, when targeting lower-limb muscles, hot-water immersion can provide higher changes in muscle temperature than hot air and for longer sessions, as the upper body promotes heat dissipation, avoiding severe hyperthermia (Fig. 2). For example, hot water temperature between 40 and 46 °C can elevate muscle temperature from 37 to 40 °C, depending on the heat exposure duration (see Table 1 from Rodrigues et al. 2022).

Although increases in muscle temperature are determinant to induce musculoskeletal adaptations, repeated elevations in core temperature may enhance neuromuscular performance. Studies involving systemic hyperthermia (i.e., increases in both core and muscle temperature) have reported temporary decreases in MVC torque and voluntary activation level (Morrison et al. 2004; Périard et al. 2014; Racinais et al. 2017; Thomas et al. 2006; Todd et al. 2005). These impairments are attributed to reduced central neural drive to the working muscles (Racinais and Oksa 2010); however, voluntary activation and muscle force return to initial levels once the core temperature cools (Morrison et al. 2004; Rodrigues et al. 2023; Thomas et al. 2006). These acute reductions in voluntary activation may lead to a supercompensation effect after repeated heat exposure, as demonstrated by Racinais et al. (2017). Following eleven consecutive days of passive heating in an environmental chamber (44–50 °C, 50% RH) targeting core temperature at 39 °C for 1 h, their study observed increases in MVC torque. Suggesting that increasing core temperature during passive heating may be important to enhance voluntary contraction performance via central nervous system adaptations.

Considering these findings, hot-water immersion appears particularly efficient for inducing high muscle temperatures while allowing for core temperature adjustments based on the body surface area immersed. This adaptability makes hot-water immersion a versatile and effective approach for achieving both localized and systemic heat stress.

## Summary and final considerations

Research into passive heat therapy is still in its infancy. While many studies have demonstrated the health benefits of passive heating, including its effects on muscle mass, recovery and neuromuscular function, limited data exist on the optimal heat dose, duration, type, and frequency for various outcomes. Therefore, we suggest incorporating the FITT principles from exercise science into thermal therapy prescription and research to help standardise protocols, guide future studies, and identify the most effective interventions for specific physiological outcomes and populations. We acknowledge that measuring thermal strain responses, such as core and intramuscular temperature, is not always feasible, accessible, or convenient for some research or laboratories. However, acknowledging this limitation and interpreting outcomes based on the level of thermal strain remains paramount. Considering the FITT framework when interpreting study findings can reduce the risk of oversimplifications and misinterpretations, such as the binary notion that “passive heat therapy does, or does not, work.”

Current evidence supports the validity of the term "exercise mimetic" for passive heat therapy, as it can elicit similar health benefits to exercise. However, passive heating sessions may require longer durations due to slower acute physiological responses than exercise. To effectively mimic exercise, passive heating interventions should aim to elicit comparable physiological responses, such as increases in muscle and core temperature, sweating rate, heart rate, and VO_2_. Accordingly, both thermal stress and physiological strain responses should be closely monitored and clearly reported. Emerging evidence also suggests that the "area under the curve" (the cumulative thermal load over time) is a critical factor for achieving positive outcomes.

Among passive heating methods, hot air types, such as saunas, have shown significant systemic health benefits, including a reduced risk of hypertension, cardiovascular and respiratory diseases, metabolic disorders, and dementia, as well as improved mental well-being and longevity (Patrick and Johnson 2021; Laukkanen and Kunutsor 2024). However, for musculoskeletal adaptation and neuromuscular function, hot-water immersion seems more efficient, as it can provide well-controlled heat stress, simultaneously inducing higher muscle temperature and adjusting the core temperature to a target level. Although microwave diathermy devices can rapidly increase muscle temperature to higher levels, hot-water immersion can target multiple muscles.

That said, if elevating core temperature can indeed promote a supercompensation effect enhancing neuromuscular function, and if skeletal muscle adaptations from passive heating are primarily driven by increases in muscle temperature in a dose-dependent manner, it must be acknowledged that progressive overload may reach practical and safety limitations. To continue eliciting physiological adaptations, increasing the duration and intensity of passive heating will encounter a ceiling effect. Therefore, future studies are warranted to better understand the nature of these thermal adaptations, and the application of FITT principles may help to establish and optimise this thermal ceiling.

## Data Availability

There is no data associated with this paper.

## Abbreviations

Akt–mTOR: Activation of the protein kinase B -mechanistic target of rapamycin
AMPK: AMP-activated protein kinase
Bpm: Beats per minute
FITT: Frequency, intensity, time, and type
HSP: Heat shock protein
MVC: Maximal voluntary contraction
NF-ĸB: Nuclear factor-ĸB
RH: Relative humidity
VO_2_: Volume of oxygen uptake
